# Advantage of Applying OSC to ^1^H NMR-Based Metabonomic Data of Celiac Disease

**DOI:** 10.5812/ijem.3058

**Published:** 2012-06-30

**Authors:** Mostafa Rezaei-Tavirani, Fariba Fathi, Fatemeh Darvizeh, Mohamad Reza Zali, Mohamad Rostami Nejad, Kamran Rostami, Mohsen Tafazzoli, Afsaneh Arefi oskouie, Seyed AbdolReza Mortazavi-Tabatabaei

**Affiliations:** 1 Proteomics Research Center, Faculty of Paramedical Sciences, Shahid Beheshti University of Medical Sciences, Tehran, IR Iran; 2 Department of Chemistry, Sharif University of Technology, Tehran, IR Iran; 3 Department of Medicine, Debrecen Medical School, Debrecen, Hungary; 4 Research Center for Gastroenterology and Liver Disease, Shahid Beheshti University of Medical Sciences, Tehran, IR Iran; 5 Acute Medicine, Dudley Group of Hospital, Dudley, UK; 6 Department of Basic Science Faculty of Paramedical Sciences, Shahid Beheshti University of Medical Sciences, Tehran, IR Iran

**Keywords:** Magnetic Resonance Spectroscopy, Principle Component Analysis, Discriminant Analysis, Celiac Disease

## Abstract

**Background:**

Celiac disease (CD) is a disorder associated with body reaction to gluten. After the gluten intake, an immune reaction against the protein occurs and damages villi of small intestine in celiac patients gradually.

**Objectives:**

The OSC, a filtering method for minimization of inter- and intra-spectrometer variations that influence on data acquisition, was applied to biofluid NMR data of CD patients.

**Patients and Methods:**

In this study, metabolites of total 56 serum samples from 12 CD patients, 15 CD patients taking gluten-free diet (GFD), and 29 healthy cases were analyzed using nuclear magnetic resonance (NMR) and associated theoretical analysis. Employing ProMetab (version ProMetab_v3_3) software, data obtained from NMR spectra were reduced and orthogonal signal correction (OSC) effect on celiac disease metabonomics before and after the separation by principle component analysis (PCA) was investigated.

**Results:**

The three groups were separated by OSC and findings were analyzed by partial least squares discriminant analysis (PLS-DA) method. Root mean square error of calibration (RMSEc) and correlation coefficient of calibration (Rc) for PLS-DA referred to an efficient group separation filtered by OSC.

**Conclusions:**

The applied leave-one-out cross-validation to PLS-DA method performed along with OSC confirmed validation of data analysis. Finally four metabolites are introduced as CD biomarkers.

## 1. Background

Celiac desease (CD) is a disorder caused essentially by body reaction to the gluten. Intake of foods containing gluten promotes an immune reaction against the protein that damages villi of small intestine in celiac patients gradually. Consequently, lack of vitamins, minerals, and other essential nutrients occurs. Therefore, celiac patients are at the risk of malnutrition, anemia and osteoporosis from which the anemia attributes to iron deficiency and results in declining red blood cell efficiency, and the osteoporosis may represent as fragile bones caused by lack of calcium (1).

Full analysis of a living organism can be achieved by an integrated set of ‘omics’ approaches including metabonomics, genomics, transcriptomics, and proteomics in order to increase data complexity. Metabonomics generates sufficient quantitative or qualitative metabolic data for analytical studies of biological systems (2-7) and is originally defined as ‘the quantitative measurement of the dynamic multi-parametric metabolic response of living systems to pathophysiological stimuli or genetic modification’(5).

Investigation of complex metabolic systems such as disease mechanisms, toxic reactions, and genetic manipulations requires full analytical data sets. Nuclear magnetic resonance (NMR) and mass spectrometry (MS) are the most conventional techniques for metabolic profiling (8-10).

Multivariate statistical approaches are also suitable methods for extraction metabolic data associated with each level of dynamic processes. Since NMR method produces various and highly correlated data, employing multivariate methods such as PCA prior to any further data analysis is essential to choose efficient number of descriptors (11).

## 2. Objectives

In this study OSC, a filtering method for minimization of inter- and intra-spectrometer variations that influence on data acquisition, was applied to biofluid NMR data of CD patients.

## 3. Patients and Methods 

### 3. 1. Blood Samples

Using syringes, two milliliters of blood samples were drawn from antecubital vein of each case through a single puncture followed by immediate serum separation via centrifugation, and storage at -20ºC. Experimental data was obtained by placing serum samples in 5mm NMR tubes. 600 µL serum samples each were diluted by 100 µL D_2_O to provide a field-frequency lock.

The experimental data consisted of 56 serum samples including 12 samples from celiac patients on gluten-free diet, 15 samples from patients without a specific diet, and 29 samples from healthy cases.

### 3. 2.^1^H NMR Spectroscopy

A BrukerAvance DRX 600 spectrometer operating at 500 MHz at 300 K using the Carr–Purcell–Meiboom–Gill (CPMG) spin–echo sequence (12) with pre saturation was employed for recording ^1^H NMR spectra. QNP probe was used in this experiment.

Spin–echo loop time (2*n*t) and relaxation delay for recorded spectra were 43.9 ms and 2s, respectively. A total of 128 transients were collected into 32k data points using a spectral width of 8389.26 Hz and an acquisition time of 1.95 s. Prior to Fourier transformation, an exponential line broadening function of 0.30 Hz was applied to the Free Induction Decay (FID).

### 3.3. Data Pre-Processing

Employing Prometab software (version Prometab_v3_3), correction of baseline was carried out according to 4, 4-Dimethyl-4-Silapentane-1-Sulfonic acid (DSS) as reference. The number of variables was reduced to 245 in each spectrum by integrating spectral intensity in regions of equal width [0.04 parts per million (ppm) over the range 0.2–10.0 ppm]. In addition, the region of (*δ* = 4.50–5.98) was excluded from the analysis to avoid unauthentic effects of variability in the suppression of water resonance. Therefore, the number of variables was reduced to 205. All spectra were normalized to constant total intensity.

### 3.4. Statistical Analysis

#### 3.4.1. OSC for Classification Modeling

A response matrix (*Y*) describing variation between defined sample classes was constructed and the variation in *X* orthogonal to *Y* was removed by applying OSC. The filtered data matrix, ***X***_osc_, that included class-correlated variations was modeled by subsequent multivariate modeling methods such as PCA or PLS-DA (14). OSC Modeling eliminates variables that are not correlated with desirable features from ^1^H NMR biofluid data; as a result calculated values in multivariate models motivate only class separation. In this study, OSC was applied to biofluid NMR data prior to chemometric analysis to minimize influences of inter- and intra-spectrometer variations during data acquisition. Moreover, it was employed to eliminate physiological variation from data sets (13-17).

#### 3.4.2. Principal Components Analysis (PCA)

PCA is a conventional technique for multivariate analysis and mainly employed for multivariate data representation in a low-dimensional space that means it describes maximum possible number of variables with minimum possible number of principal components (PCs). In PCA, principal components are named consecutively starting from PC_1_ until total variance is defined. PC_1_ or first principal component is a line that goes through the points in a variable space and best conserves relevant distances between objects and defined by a loading vector as follows. PC_1_ is a hidden variable and has maximum variance of the scores. Scores are predicted data values on the hidden variable (18).

All calculations were performed in MATLAB 6.5 and PCA was implemented with the PLS-Toolbox Version 3.0. Both the graphical (‘pcagui’) and the command-line (‘pca’) versions of PCA have been employed (19).

#### 3.4.3. Partial Least Squares Discriminant Analysis (PLS-DA)

PLS regression was directed by a response data set Y to derive components from descriptor data set X that best describe specified Y structure, as it maximizes the covariance that expresses common structure between X and Y (20-22). PLS is also divided into regression as well as discriminant analysis (PLS-DA). Classification by DA assigns the samples to proper separate classes which are represented by using so-called ‘dummy’ variables (23).

## 4. Results

**Figure 1:**
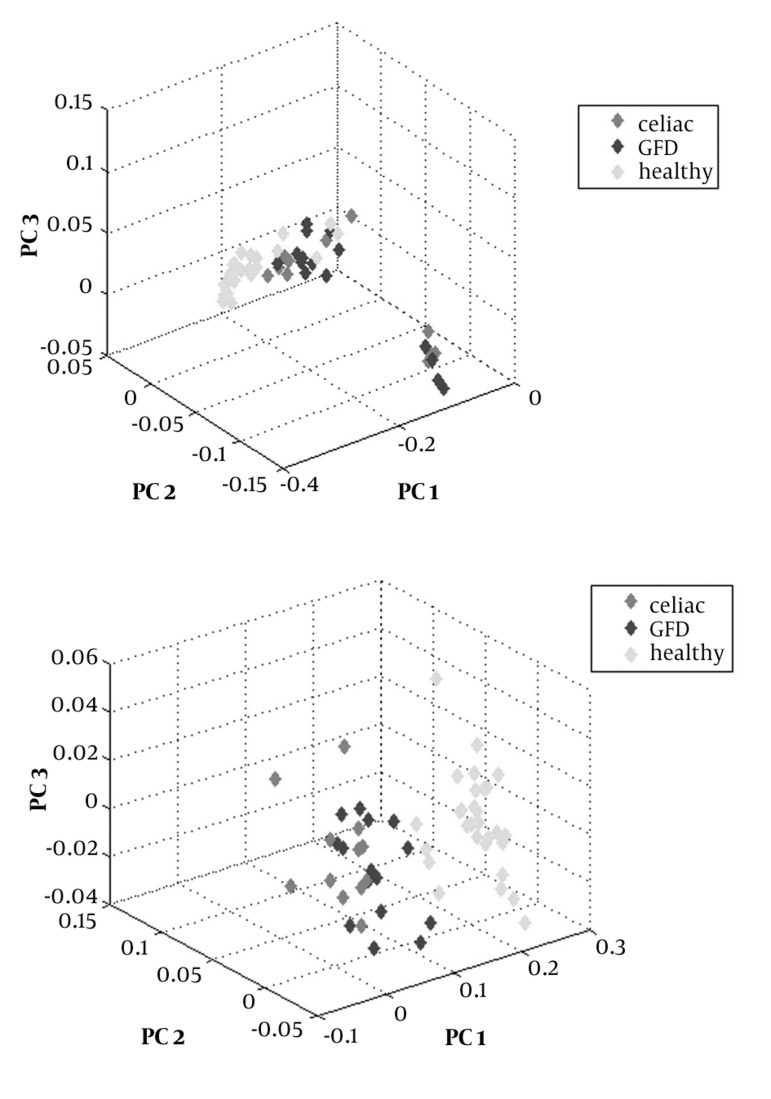
Score Plot PCA a) Without OSC, and b) With OSC

As shown in Figure 1a, data analysis leads to introduce two groups of control and celiac cases. Since separation of GDF group from other two groups was an important index in this study, more analytical efforts were effected by using OSC in order to achieve better transparency between sample groups. The useful role of OSC in separation groups is reported in several studies (24). By using OSC, 95.26% total variability was distributed between three PCs as PC_1_ (91.11%), PC_2_ (2.88%), and PC_3_ (1.47%) (See Figure 1b). After successful division of samples into three groups, PLS-DA as regression extension of PCA was applied to maximize the separation. It is reported that PLS-DA is useful tool for maximizing covariance between measured data (x) and response variable (y) (25). PLS-DA findings introduced three most influential metabolites that play important role in separation of groups. Serum level alteration of these metabolites in CD group compared to control group is shown in Table 1.

**Table 1:** PCA-Detected ^1^H NMR Spectral Regions that Separate Significantly CD and Control Groups Based on Metabolites Levels

Chemical Shift (ppm)	Metabolite	Alteration of Metabolite Level in CD Group Compared to Control Group
0.86	lipid (mainly VLDL)	Decrease
1.26 -1.3	Lipid	Decrease
1.34	Lactate	Decrease
3.22	Choline	Increase

The percentage of captured variance by PCA model is a suitable index for data validation (26); accordingly, three times PCs and their associated variance amounts were calculated and shown in Table 2 with total variance equal to 92.22% out of which the percentage variance in step 1 was equal to 84.38%, and in second and third steps were 4.74 and 3.12, respectively. Since 92.24% is close to 1, we may conclude that PCA model could separate the three groups in a real and valid manner.

**Table 2:** Percent Variances Captured by PCA Model. First column corresponds to steps of model application and the second and third columns refer to variance of each step and total variance, respectively.

Number	% Variance	% Variance Total
1	84.38	84.38
2	4.74	89.12
3	3.12	92.24

**Figure 2:**
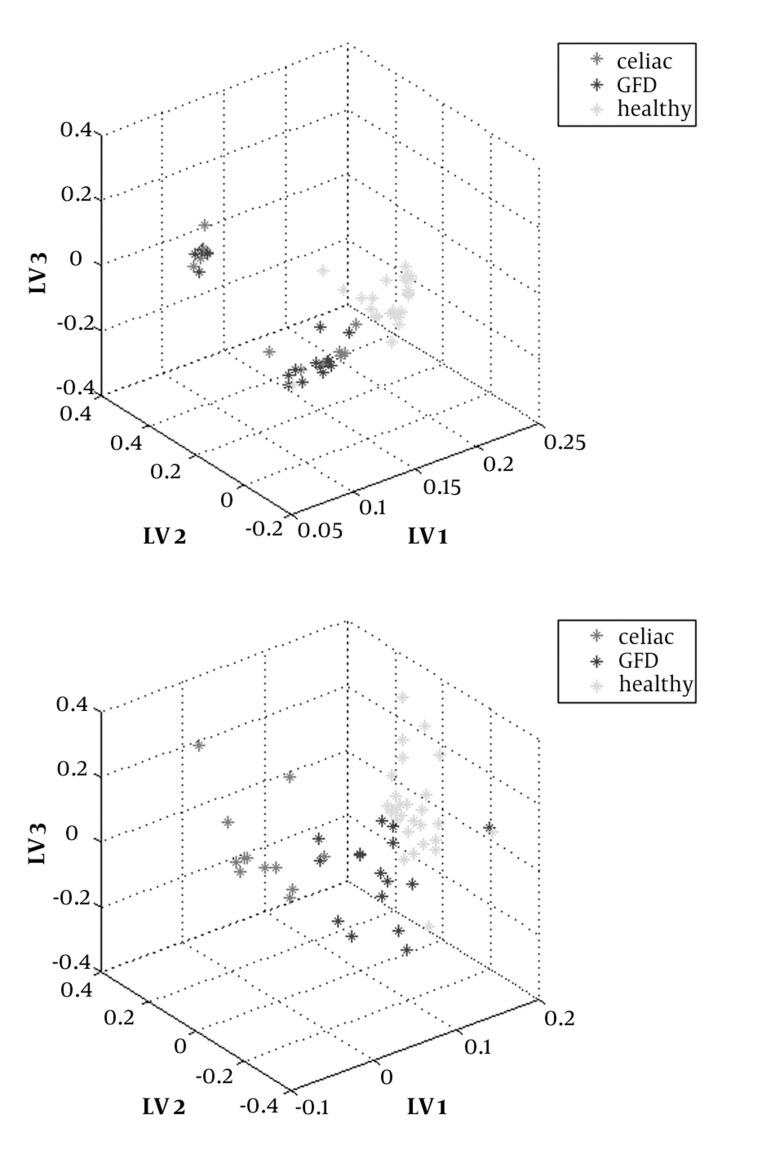
Score Plot PLS-DA a) Without OSC, and b) With OSC

Score plots of PLS-DA without and with application of OSC for three groups are shown in Figure 2. The useful parameter to analyze PLS-DA findings is latent variable variance (LV) (16). LVs of represented data in Figure 2 for three steps of PLS-DA were calculated and shown in Table 3. There are several evidences implicate that total variance value above 80% is an acceptable index for data validation. Accumulated PLS-DA score plot by using OSC for three LV variances was 86.82% (see Table 3) that revealed a clear separation between the three groups; however this procedure without applOSC application (identified by an accumulated score plot of 75.55%) could not be considered as a suitable analytical method.

**Table 3:** Summary of PLS-DA and OSC- PLS-DA Models of NMR Spectra. LVs refer to number of latent variances and Vx and Vy correspond to NMR data matrix and respond matrix, respectively. Vx (cuml, %) and Vy show total variances.

LV_s_	V_X_ (%)	V_X_ (cuml, %)	V_Y_ (%)	V_Y_ (cuml, %)
**PLS-DA Model**				
1	63.96	63.96	44.07	44.07
2	9.05	73.01	16.34	60.41
3	2.54	75.55	8.24	66.73
**OSC- PLS-DA Model**				
1	75.25	75.25	100.00	100.00
2	6.11	81.36	0.00	100.00
3	5.46	86.82	0.00	100.00

RMSEc and Rc values for PLS-DA calibration without applying OSC were equal to 0.6682 and 0.6348, respectively. By using OSC, these values changed to 0.4226 and 0.8629, respectively. Amounts of Rc close to 1 correspond to acceptable data analysis (27) that means the calculated Rc value of 0.8629 refers to application of a proper analytical method for group separation. Besides this, OSC application reduced RMSEc value, that means an error reduction occurred. For better clarity, the applied OSC model was performed accompanied by leave-one-out cross-validation. The characterized findings were 0.5175 and 0.8510 for RMSEcv and Rcv, respectively. The low amount of error and Rcv close to 1, confirm sufficient validity of findings.

## 5. Discussion

According to orthogonal theory in mathematics, OSC can eliminate data in X matrix that are orthogonal with response Y matrix. The response Y matrix is class variable. The Y matrix variables determined as control, CD, and GFD groups were assigned to as 0 , 1, and 2, respectively. Orthogonal component with eigenvalues greater than 1 were eliminated. In order to perform metabonomics analysis of recorded ^1^H NMR spectra of healthy, celiac, and GFD groups, two different recognition pattern methods before and after OSC were applied. Score plots of PCA are drawn using the MATLAB software. Figure 1a and Figure 1b demonstrate PCA score plots of NMR spectra without and with OSC, respectively.

On the basis of this study, four metabolites were introduced to differentiate between celiac patients on GFD, celiac patients without specific diet, and healthy people. We hope that further investigations lead to determine exact metabonomic pattern of CD.
